# Osteoinduction on Acid and Heat Treated Porous Ti Metal Samples in Canine Muscle

**DOI:** 10.1371/journal.pone.0088366

**Published:** 2014-02-10

**Authors:** Toshiyuki Kawai, Mitsuru Takemoto, Shunsuke Fujibayashi, Haruhiko Akiyama, Masashi Tanaka, Seiji Yamaguchi, Deepak K. Pattanayak, Kenji Doi, Tomiharu Matsushita, Takashi Nakamura, Tadashi Kokubo, Shuichi Matsuda

**Affiliations:** 1 Department of Orthopedic Surgery, Graduate School of Medicine, Kyoto University, Kyoto, Japan; 2 Department of Biomedical Sciences, College of Life and Health Sciences, Chubu University, Kasugai, Aichi, Japan; 3 Osaka Yakin Kogyo Co., ltd., Osaka, Japan; 4 National Hospital Organization Kyoto Medical Center, Kyoto, Japan; University of Zurich, Switzerland

## Abstract

Samples of porous Ti metal were subjected to different acid and heat treatments. Ectopic bone formation on specimens embedded in dog muscle was compared with the surface characteristics of the specimen. Treatment of the specimens by H_2_SO_4_/HCl and heating at 600°C produced micrometer-scale roughness with surface layers composed of rutile phase of titanium dioxide. The acid- and heat-treated specimens induced ectopic bone formation within 6 months of implantation. A specimen treated using NaOH followed by HCl acid and then heat treatment produced nanometer-scale surface roughness with a surface layer composed of both rutile and anatase phases of titanium dioxide. These specimens also induced bone formation after 6 months of implantation. Both these specimens featured positive surface charge and good apatite-forming abilities in a simulated body fluid. The amount of the bone induced in the porous structure increased with apatite-forming ability and higher positive surface charge. Untreated porous Ti metal samples showed no bone formation even after 12 months. Specimens that were only heat treated featured a smooth surface composed of rutile. A mixed acid treatment produced specimens with micrometer-scale rough surfaces composed of titanium hydride. Both of them also showed no bone formation after 12 months. The specimens that showed no bone formation also featured almost zero surface charge and no apatite-forming ability. These results indicate that osteoinduction of these porous Ti metal samples is directly related to positive surface charge that facilitates formation of apatite on the metal surfaces in vitro.

## Introduction

Various types of porous materials have been found to exhibit osteoinduction, which is ectopic bone formation on a material without the addition of living cells and/or growth factors such as bone morphogenetic proteins. Although the exact mechanisms of osteoinduction by materials remain largely unknown, there is considerable interest for potential clinical applications.

Most of the materials in which osteoinduction has been found are based on calcium phosphate [1]–[6]. However, Fujibayashi found that porous titanium metal with no calcium phosphate can also exhibit osteoinduction, if it is subjected to certain chemical and heat treatments [7]. A porous titanium (Ti) metal specimen produced by plasma-spray deposition exhibited osteoinduction when embedded in dog muscle, following: NaOH and heat treatments; NaOH and water treatments; NaOH, HCl and water treatments [8]. Untreated samples did not exhibit osteoinduction. The degree of osteoinduction was greatest for the NaOH, HCl and water treated samples and the NaOH treated samples exhibited the least growth. All of the porous Ti metal subjected to these chemical and heat treatments also showed apatite formation on their surfaces in a simulated body fluid (SBF) with ion concentrations almost equal to those of the human blood plasma [8]. These results indicate that osteoinduction is facilitated by the formation of apatite on the metals surfaces in vivo, as has been speculated for other osteoinductive materials [9].

Porous Ti metal produced by sintering of a Ti fiber mesh did not exhibit any osteoinduction, even when subjected to NaOH and water treatments, although apatite formation occurred on its surface in SBF [7]. This result indicates that osteoinduction of porous Ti metals specimens depends not only upon the formation of apatite on the sample surface, but also the morphological characteristics of the porous structure.

In recent studies we found that Ti metal specimens subjected to a simple acid treatment followed by heat treatment without an initial NaOH treatment also formed apatite on their surface in SBF [10]. The Ti metal specimen that was acid and then heat treated showed micrometer-scale roughness [10], [11], whereas a specimen treated by NaOH and then heat-treated showed nanometer-scale surface roughness [12]–[15]. The Ti metal specimen that was acid and then heat treated was found to be strongly bonded to living bone, when it was implanted into rabbit tibia in the form of a rectangular plate [11], and was deeply penetrated with newly grown bone when it was implanted into rabbit femur as a porous structure [16]. It was speculated that the heating of the porous Ti metal specimen after the acid treatment altered the samples to exhibit osteoinduction.

In the present study, osteoinduction of acid and heat treated porous Ti metal specimens in dog muscle was examined, and compared with the results from specimens subjected to only acid or heat treatments and with untreated specimens. We also compared the results of osteoinduction on acid and heat treated porous Ti metal specimens that underwent a NaOH pretreatment. The osteoinduction results were related to the surface charge, degree of apatite-formation in SBF, and the morphological characteristics of the Ti metal specimens.

These studies develop a fundamental understanding of osteoinduction processes in porous Ti metal. The findings also have relevance to the clinical applications of such materials as load-bearing bone substitute materials.

## Materials and Methods

### Sample Preparation

Ti metal powder with 45-µm mesh size was obtained from Osaka Yakin Kogyou Co., Japan. Ammonium hydrogen carbonate was obtained from Kishida Chemical Co., Japan and passed through 1400- and 500-µm meshes before use. These powders were mixed, uniaxially pressed at 100 MPa and then sintered at 1400°C for 2 h in an argon atmosphere, to obtain a porous Ti metal body. Full details for preparation of the porous body have been described elsewhere [16]–[18]. Cylindrical specimens 6 mm in diameter and 15 mm in length were cut from the sintered porous body. These were washed with acetone, 2-propanol and ultrapure water for 30 min each using an ultrasonic cleaner, and then dried overnight in an oven at 40°C.

Six cylindrical specimens were used directly without further treatment, two for material characterization (one specimen for structural analysis and another for observation of inner surface) and the remaining four specimens were used in animal experiments, as described in the following sections.

Another six cylindrical specimens were heat treated by heating to 600°C at a rate of 5°C min^−1^, kept at 600°C for 1 h, and then allowed to cool naturally to room temperature in an iron-chromium furnace.

Twelve cylindrical specimens were subjected to a mixed acid treatment by soaking in 20 mL of 1∶1(w/w) mixture of 66.3% H_2_SO_4_ (w/w) solution (Kanto Chemical Co., Inc) and 10.6% HCl (w/w) solution (Kanto Chemical Co., Inc.) at 70°C for 30 min in an oil bath shaken at 120 strokes min^−1^. They were gently washed with ultrapure water and dried overnight in an oven at 40°C.

Six of the 12 mixed acid-treated specimens were subsequently subjected to the same heat treatment as described above.

Another 12 cylindrical specimens were first subjected to NaOH treatment, by soaking in 5 mL of 5.0 M NaOH solution at 60°C for 24 h. These were then subjected to an acid treatment by soaking in either 0.5 mM (six specimens) or 50 mM HCl solution (six specimens) at 40°C for 24 h, and then finally subjected to the same heat treatment as described above.

The specimens that were fabricated and their treatments are summarized in Table 1.

**Table 1:** Treatments, structure and properties of porous Ti metal specimens.

Sample name	Treatment	Surfacetexture	Phasebesides Ti	Contact angle(degree)	Zeta potential(mV)	Apatiteformation	Osteoinduction
**Un**	No	Smooth	No	72.4 (±2.2)	∼0	No	No
**Ht**	Heat	Smooth	Rutile	66.4 (±1.7)	−2.1 (±3.1)	No	No
**Ac**	Mixed acid	Micro rough	Titanium hydride	71.2 (±3.0)	∼0	No	No
**Ac-Ht**	Mixed acid+heat	Micro rough	Rutile	58.8 (±2.3)	+8.0 (±2.0)	High	High
**Na-0.5H-Ht**	NaOH, 0.5 mHCl and heat	Nano rough	Anatase+Rutile	27.6 (±3.8)	+3.4 (±2.3)	Moderate	Moderate
**Na-50H-Ht**	NaOH, 50 mHCland heat	Nano rough	Anatase+Rutile	11.2 (±1.8)	+8.4 (±1.8)	High	High

### Analysis of Structure of the Specimens

The total porosity and distribution of interconnected pores of the porous Ti metal specimens prepared by the methods described above were measured by Hg vapor penetration in an evacuated porosimeter (Autopore 9420, Micrometrics, USA).

The morphology of pores and texture of the inner surfaces of the pores of the porous Ti metal were imaged at a fracture surface using a scanning electron microscope (SEM; S-4700, Hitachi Ltd., Japan).

The crystalline phases formed on the pore wall of the porous Ti metal specimens were determined by examining surfaces of Ti metal plates subjected to the same treatments as those for the porous Ti metals by thin-film X-ray diffraction (TF-XRD; RINT-2500, Rigaku Co., Japan). One plate for each treatment group was used this analysis.

### Wettability Test

Ti metal plates 10×10×1 mm^3^ in size were subjected to the same treatments as those for the porous Ti metal specimens. Contact angles of pure water on the Ti metals plates were measured by a droplet method according to a modified JIS R3257 testing method. A drop of pure water (4 µL) was gently dropped onto the metal plates and the contact angles with the sample surfaces were measured by a half angle method. This test was repeated five times on each sample.

### Zeta Potential Measurements

Ti metal plates 13×33×1 mm^3^ in size were subjected to the same treatments as those for the porous Ti metal specimens. The plates were earthed on one side to allow for leakage of any stray charge and placed in the glass cell of a zeta potential and particle size analyzer (model ELS-Z1, Otsuka Electronics Co., Japan). The zeta potentials were measured under an applied voltage of 40 V in 10 mM NaCl solution. Hydroxypropyl cellulose-coated polystyrene latex particles 500 nm in size were used as monitoring particles. Five specimens were measured for each sample, and the mean value (± standard deviation: SD) was determined and is shown in Table 1.

### Examination of Apatite-forming Ability in SBF

Cylindrical specimens of the porous Ti metals subjected to the various treatments given in Table 1 were broken and soaked in 30 mL of a simulated body fluid (SBF) having ion concentrations close to those of human blood plasma (Na^+^ = 142.0, K^+^ = 5.0, Mg^2+^ = 1.5, Ca^2+^ = 2.5, Cl^–^ = 147.8, HCO_3_^–^ = 4.2, HPO_4_^2–^ = 1.0, and SO_4_^2–^ = 0.5 mM) [19], [20] at 36.5°C. The SBF was prepared by dissolving reagent-grade NaCl, NaHCO_3_, KCl, K_2_HPO_4_·3 H_2_O, MgCl_2_.6 H_2_O, CaCl_2_, and Na_2_SO_4_ (Nacalai Tesque Inc., Japan). After 1 day, the specimens were removed from the SBF solution, gently washed with ultrapure water and dried in an oven at 40°C. Formation of apatite on their surfaces was examined by SEM and TF-XRD analysis.

### Animal Experiments

The cylindrical specimens of the porous Ti metals specimens subjected to the various treatments (Table 1) were used in the following animal experiments. These experiments were carried out in strict accordance with the recommendations in the Guide for the Care and Use of Laboratory Animals of the National Institutes of Health. The protocol was approved by the Animal Research Committee, Graduate School of Medicine, Kyoto University, Japan (Permit Number: Med Kyo 10286). All surgery was performed at Graduate School of Medicine, Kyoto University. All efforts were made to minimize suffering.

The specimens were sterilized with ethylene oxide gas and implanted into the dorsal muscles of four beagle dogs (weight, 10–11 kg) for periods of 6 or 12 months. The owner of the dogs (Graduate School of Medicine, Kyoto University) gave permission for the animals to be used in this study. The animals were anesthetized by intramuscular administration of ketamine hydrochloride (50 mg/kg), followed by diazepam (5 mg) and atropine sulfate (0.5 mg) without endotracheal intubation. Just before the operation, a dose of 10 mg/kg of pentobarbital sodium was injected intravenously. The operations were performed under standard sterile conditions. After incising the skin and fascia, muscle pouches were carefully made in the dorsal muscle to limit any bleeding. Each specimen was implanted in a separate pouch, while maintaining sufficient distance (more than 6 cm) between each specimen to prevent inter-specimen contact. Each pouch was marked with 3-0 nylon to facilitate extraction. Each dog was implanted with 12 samples (two for each treatment group) and six samples were harvested (one from each treatment group) at 6 months. The remaining six samples were retrieved at 12 months. At 6 and 12 months after implantation, the animals were anesthetized by the same method used at implantation and each specimen site was retrieved.

### Histological Examination

Following extraction, the specimen sites were removed and prepared for histological examination. The specimens were fixed in 10% phosphate-buffered formalin (pH 7.25) for 7 days, and dehydrated in serial concentrations of ethanol (70%, 80%, 90%, 99%, 100%, and 100% v/v) for 3 days in each. The specimens were then embedded in polyester resin. Thick sections (250 µm) were cut with a band saw (BS-3000CP, EXACT cutting system, Norderstedt, Germany) perpendicular to the axis of the implant, and ground to a thickness of 40–50 µm using a grinding-sliding machine (Microgrinding MG-4000, EXACT). Each section was then stained with Stevenel’s blue and Van Gieson’s picrofuchsin. Microscopic analysis was performed on histological slides using transmitted light microscopy (Nikon Model Eclipse 80i) combined with a digital camera (Nikon Model DS-5M-L1).

### Histomorphometric Examination

New bone growth rate (%), which was defined as the percentage of bone area in the pore area available for bone ingrowth, was obtained on each section by using Adobe Photoshop CS 5 (Adobe System Inc., San Jose, CA, USA) and ImageJ software (National Institute of Health, Bethesda, MA, USA). The pore area available for bone ingrowth was calculated by subtracting the area of titanium metal from the total area of the specimen. Two sections were cut and examined for each sample. Each section was cut 3 mm from the end of the sample leaving a distance of 9 mm between the two sections.

### Statistics

All data herein are expressed as mean ± standard deviation (SD). They were statistically analyzed using one-way analysis of variance (ANOVA) followed by post-hoc testing (Tukey–Kramer multiple comparison test). For statistical analysis, JMP 9 software (SAS Institute, Cary, NC, USA) was used. Differences at *p*<0.05 were considered statistically significant.

## Results

### Structure of Porous Ti Metal

**Figure 1:**
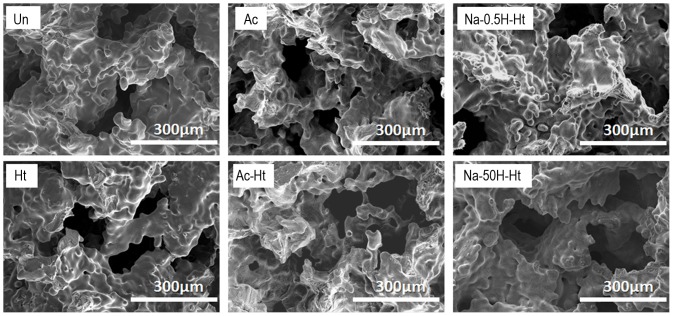
SEM imagesof fractured surfaces of the porous Ti metal specimens subjected to the various treatments described in Table 1. Un: Untreated, Ht: heat treated, Ac: Mixed acid treated, Ac-Ht: Mixed acid and heat treated, Na-0.5H-Ht: NaOH: NaOH, 0.5 mM HCl and heat treated, Na-50H-Ht: NaOH, 50 mM HCl and heat treated.

SEM images of the fracture surfaces of the various porous Ti metal specimens are shown in Fig. 1. All the samples examined contain interconnected pores 100 to 200 µm in size, which had irregular surfaces composed of sintered small particles.

**Figure 2:**
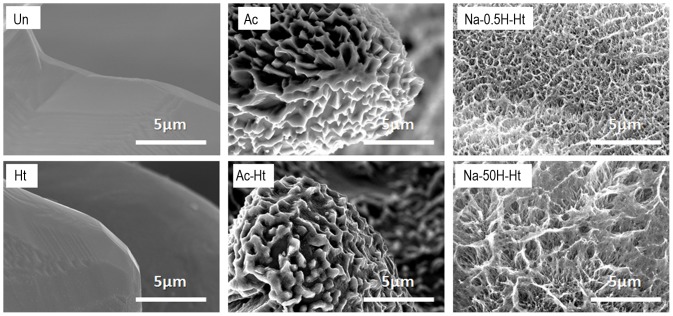
SEM images of inner walls of the pores of the porous Ti metal specimens subjected to the various treatments described in Table 1. Un: Untreated, Ht: heat treated, Ac: Mixed acid treated, Ac-Ht: Mixed acid and heat treated, Na-0.5H-Ht: NaOH: NaOH, 0. 5mM HCl and heat treated, Na-50H-Ht: NaOH, 50 mM HCl and heat treated.

SEM images of the inner wall of the pores from various specimens are shown in Fig. 2. It can be seen from Fig. 2 that the specimen subjected to only the heat treatment (Table 1, Ht) has smooth pore walls. The specimens subjected to the acid and then heat treatment (Table 1, Ac-Ht) presented with pore walls that had micrometer-scale roughness. The specimens that were pretreated with NaOH before acid and heat treatment (Table 1, Na-0.5H-Ht and Na-50H-Ht) showed pore walls with nanometer-scale roughness. The differences in surface texture of the various specimens are summarized in Table 1.

**Figure 3:**
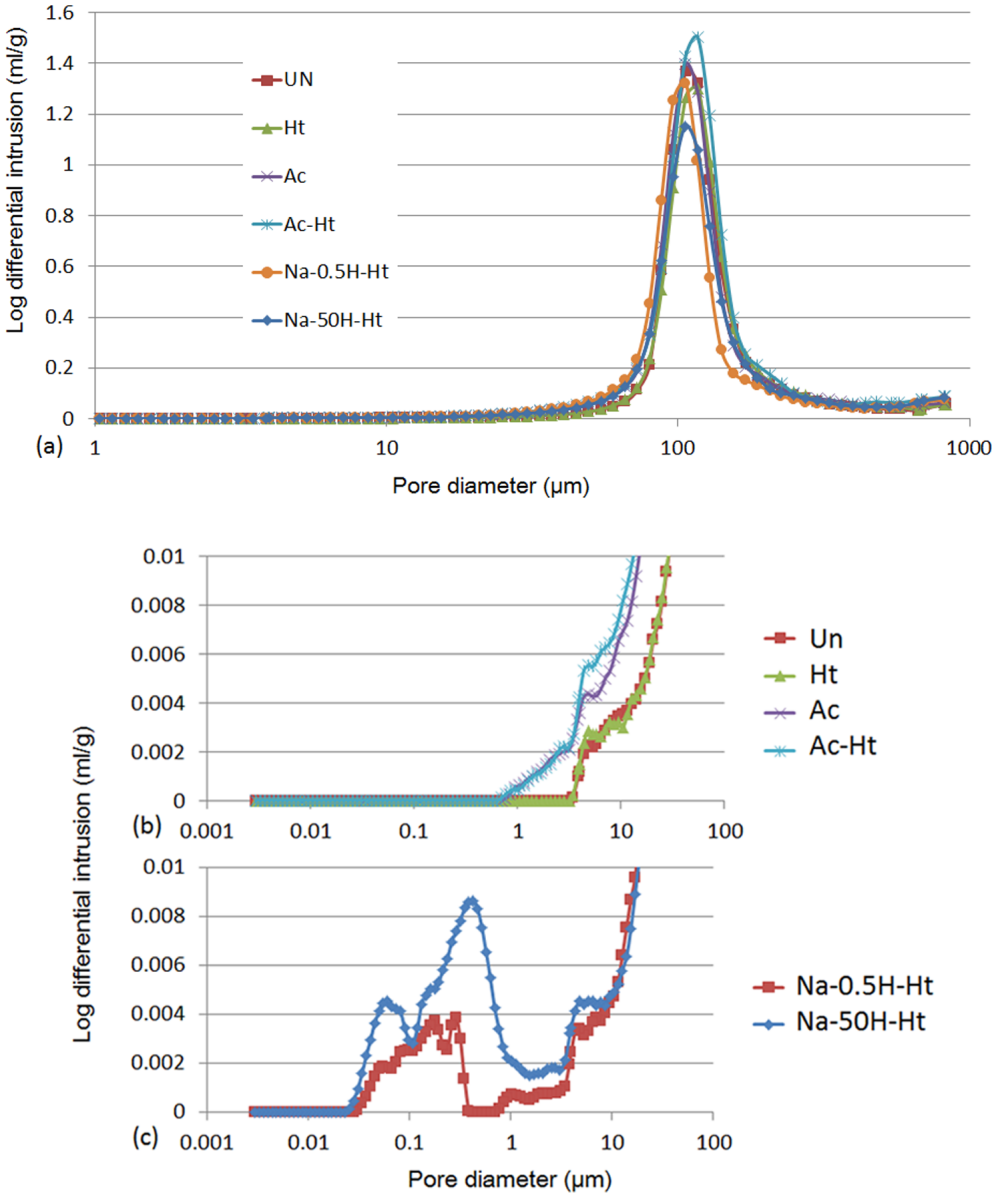
Distribution of pore diameters on a full scale (a) and expansions (b), (c) for the porous Ti metals subjected to various treatments given in Table 1, as measured by Hg penetration porosimetry, where the x-axis is pore diameter, and the y-axis is log differential volume of Hg that penetrated into the pores of the sample per unit weight. Un: Untreated, Ht: heat treated, Ac: Mixed acid treated, Ac-Ht: Mixed acid and heat treated, Na-0.5H-Ht: NaOH: NaOH, 0.5 mM HCl and heat treated, Na-50H-Ht: NaOH, 50 mM HCl and heat treated.

The volumes of the pores in the porous Ti metal specimens are shown in Fig. 3 (a), (b) and (c), determined from Hg penetration porosimetry measurements and as a function of pore diameter. All the specimens examined had micro-scale pores ranging from 70 to 200 µm in diameter, giving a total porosity of 59%, irrespective of the treatments (Fig. 3 (a)).

**Figure 4:**
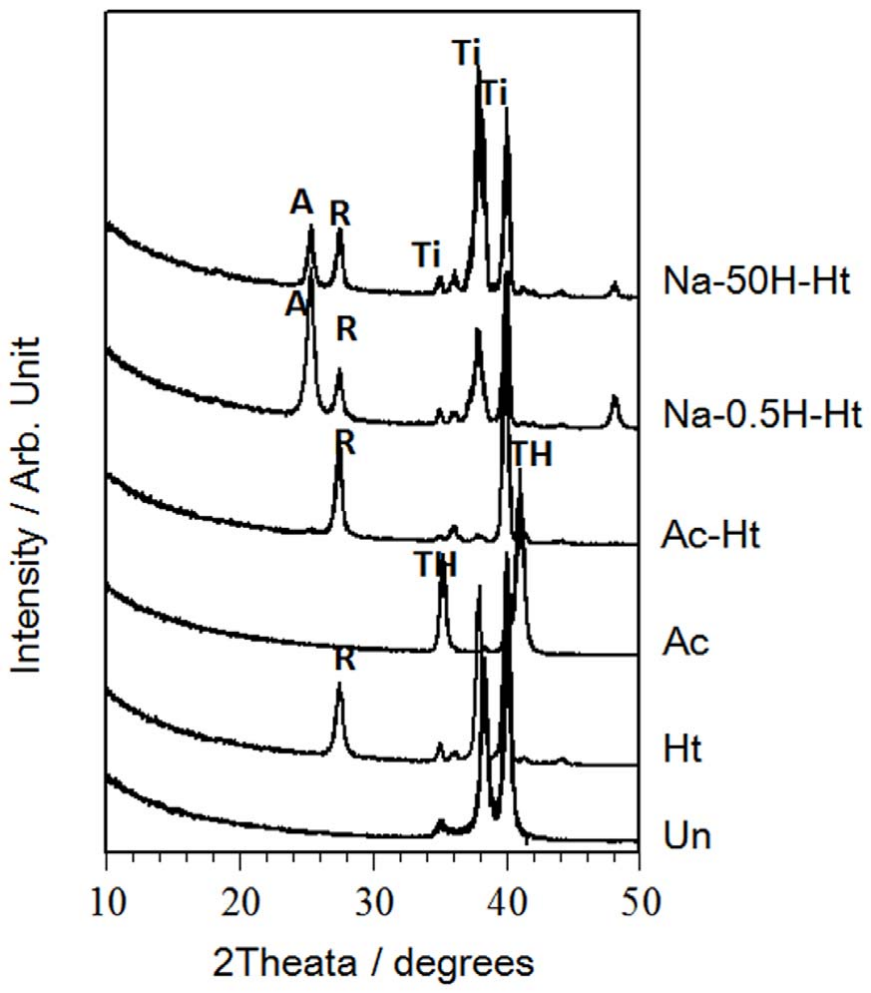
TF-XRD diffraction patterns of the surfaces of the porous Ti metal specimens subjected to various treatments described in Table 1, Un: Untreated, Ht: heat treated, Ac: Mixed acid treated, Ac-Ht: Mixed acid and heat treated, Na-0.5H-Ht: NaOH: NaOH, 0.5 mM HCl and heat treated, Na-50H-Ht: NaOH, 50 mM HCl and heat treated. Ti: Titanium, TH: Titanium hydride, R: Rutile, A: Anatase.

The porosity measurements also show that the specimens subjected to the mixed acid and heat treatment (Table 1, Ac-Ht ) also featured pores ranging from 1 to 10 µm in diameter (Fig. 3 (b)) in addition to the larger ∼100 µm-pores present in all specimens. The base, acid and heat treated specimens (Table 1, Na-0.5H-Ht and Na-50H-Ht) also showed pores ranging from 0.05 to 0.5 µm in diameter, in addition to the larger ∼100 µm-pores (Fig. 3 (c)). The TF-XRD patterns of the various Ti metal plate samples are shown in Fig. 4. The untreated sample (Table 1, Un) showed only a titanium metal phase. Heat treated samples (Table 1, Ht) showed a rutile phase of titanium oxide on its surface and titanium hydride was found after the mixed acid treatment (Table 1, Ac-Ht). The titanium hydride likely transformed into the rutile during the heat treatment. An anatase phase of titanium dioxide was detected with rutile for samples treated by NaOH, acid and heat treatments (Table 1, Na-0.5H-Ht and Na-50H-Ht). The crystalline phases assigned to the TF-XRD patterns measured for each of the specimens are summarized in Table 1.

### Contact Angle

Contact angles of pure water on the specimens are summarized in Table 1.

### Zeta Potential

Zeta potentials measured for the specimens are summarized in Table 1. The specimens that were only heat or acid treated (Table 1, Ht and Ac) showed almost zero or weak negative zeta potentials. The specimens subjected to acid and heat treatments (Table 1, Ac-Ht), and those that were pretreated with NaOH (Table 1, Na-0.5H-Ht and Na-50H-Ht) showed large positive zeta potentials.

### Apatite Formation

**Figure 5:**
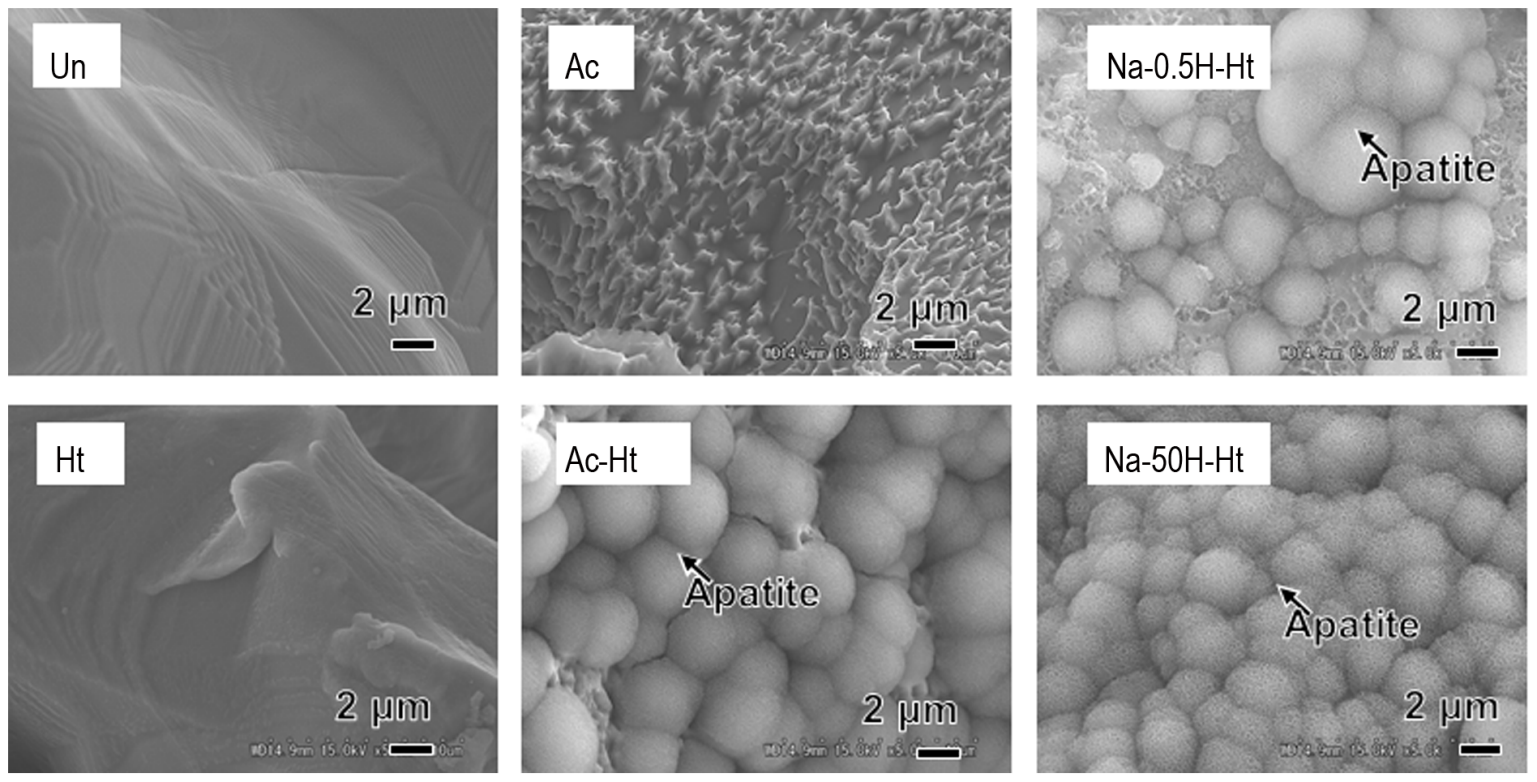
SEM images of fracture surfaces of various porous Ti metal specimens (Table 1) that were broken and soaked in SBF for 1 day. Un: Untreated, Ht: heat treated, Ac: Mixed acid treated, Ac-Ht: Mixed acid and heat treated, Na-0.5H-Ht: NaOH: NaOH, 0.5 mM HCl and heat treated, Na-50H-Ht: NaOH, 50 mM HCl and heat treated.

SEM images of the surface of the porous Ti metals are shown in Fig. 5. The specimens were fractured and soaked in SBF for 1 day. It can be seen from Fig. 5 that apatite formed on the surface of the specimens subjected to mixed acid and heat treatments (Table 1, Ac-Ht) and NaOH, acid and heat treatments (Table 1, Na-0.5H-Ht and Na-50H-Ht), but not on untreated (Table 1, Un) and only heat or acid treated specimens (Table 1, Ht and Ac). The degree of apatite formation on each specimen is also summarized in Table 1.

### Histological Findings

All the dogs examined tolerated the surgical procedure well. They showed neither infection at the surgical site, dislocation of the implants, nor adverse reaction to the foreign body such as inflammation around the implants.

**Figure 6:**
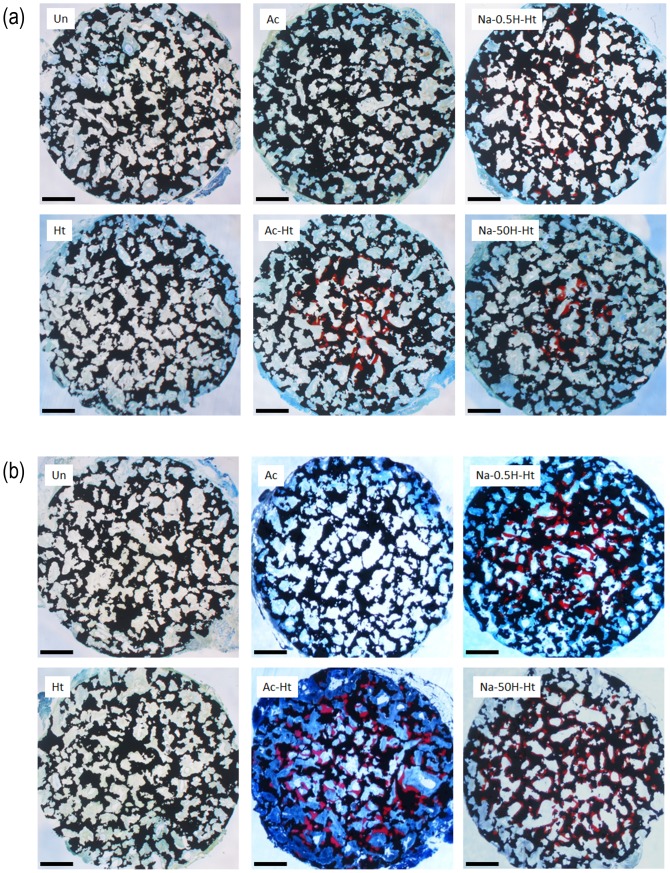
Optical microscope images of non-decalcified histological sections of porous Ti metals subjected to various treatments given in Table 1, after implantation in dog back muscle for 6 (a) and 12 (b) months. Scale bar: 1 mm. Un: Untreated, Ht: heat treated, Ac: Mixed acid treated, Ac-Ht: Mixed acid and heat treated, Na-0.5H-Ht: NaOH: NaOH, 0.5 mM HCl and heat treated, Na-50H-Ht: NaOH, 50 mM HCl and heat treated.

**Figure 7:**
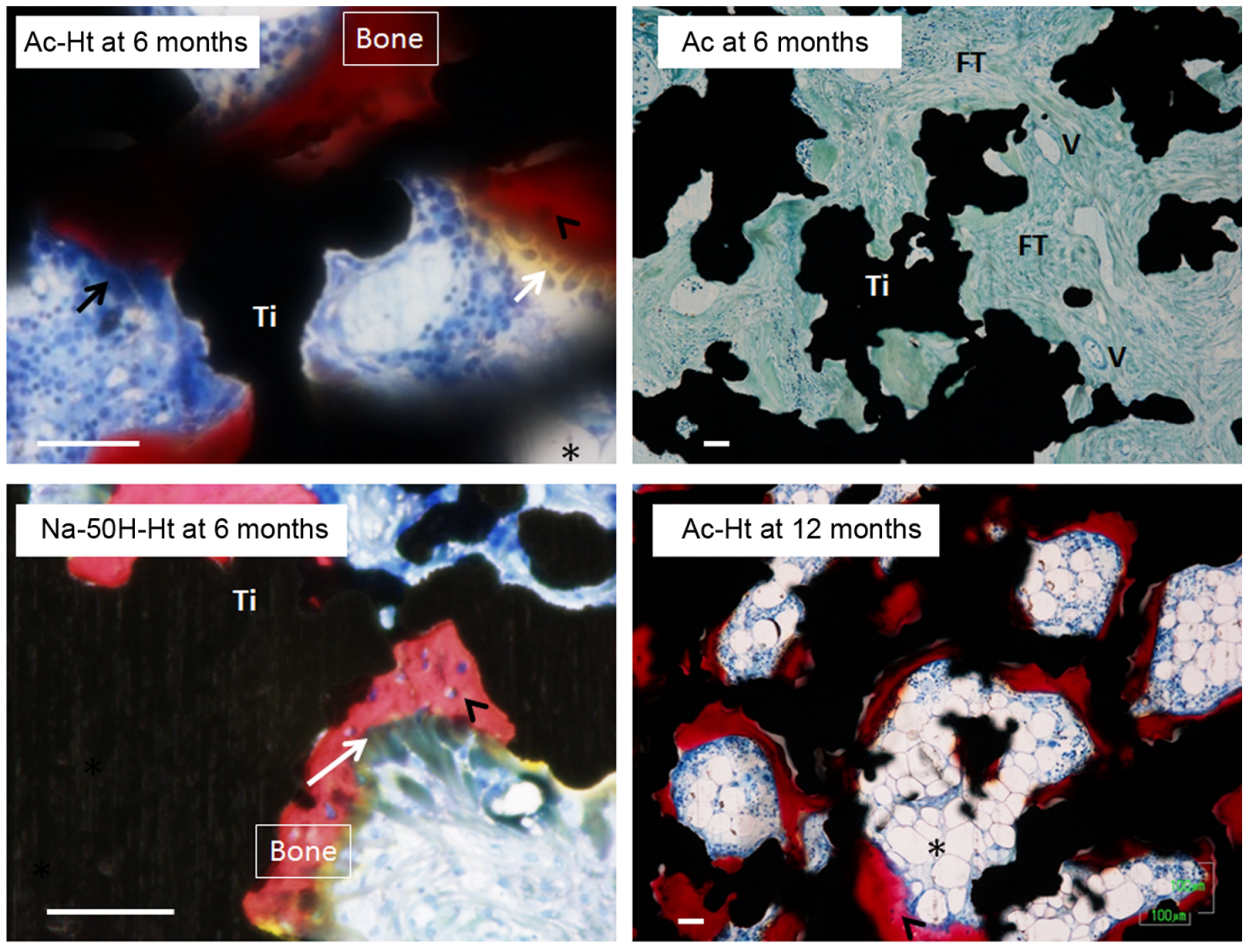
High magnification optical microscope images of non-decalcified histological sections given in Fig. 6. Scale bar: 50 µm. Asterisk: marrow-like formation. White arrow: cuboidal osteoblast-like cells. Black arrow: osteoclast-like multinucleated cells. Arrow head: osteocyte. Un: Untreated, Ht: heat treated, Ac: Mixed acid treated, Ac-Ht: Mixed acid and heat treated, Na-0.5H-Ht: NaOH: NaOH, 0.5 mM HCl and heat treated, Na-50H-Ht: NaOH, 50 mM HCl and heat treated.

Optical micrographs of non-decalcified histological sections of the porous Ti metal specimens are shown in Fig. 6 (a) and (b), after implantation in the back muscles of beagle dogs for 6 and 12 months, respectively. High magnification optical microscope images of the non-decalcified histological sections are shown in Fig. 7.

The figures show that the porous Ti metals subjected to the mixed acid and heat treatments (Table 1, Ac-Ht), and to NaOH, 0.5 mM HCl or 50 mM HCl and heat treatments (Table 1, Na-0.5H-Ht and Na-50H-Ht) formed a large amount of bone containing osteocytes in the central part of their porous structures at 6 months after implantation. The bone growth spread to the periphery of the porous structures 12 months after the implantation (Fig. 6(b)). Marrow-like tissue and cuboidal osteoblast-like cells were also observed around the new bone, and osteoclast-like multinucleated cells were observed on the new bone (Fig. 7).

Conversely, the untreated specimens (Table 1, Un) and those subjected to only heat or only acid treatments (Table 1, Ht and Ac) showed no bone formation even 12 months after the implantation. Fibrous connective tissue and newly formed blood vessels were observed in the pore regions of the non-osteoinductive samples (Fig. 7).

### New Bone Growth Rate

**Figure 8:**
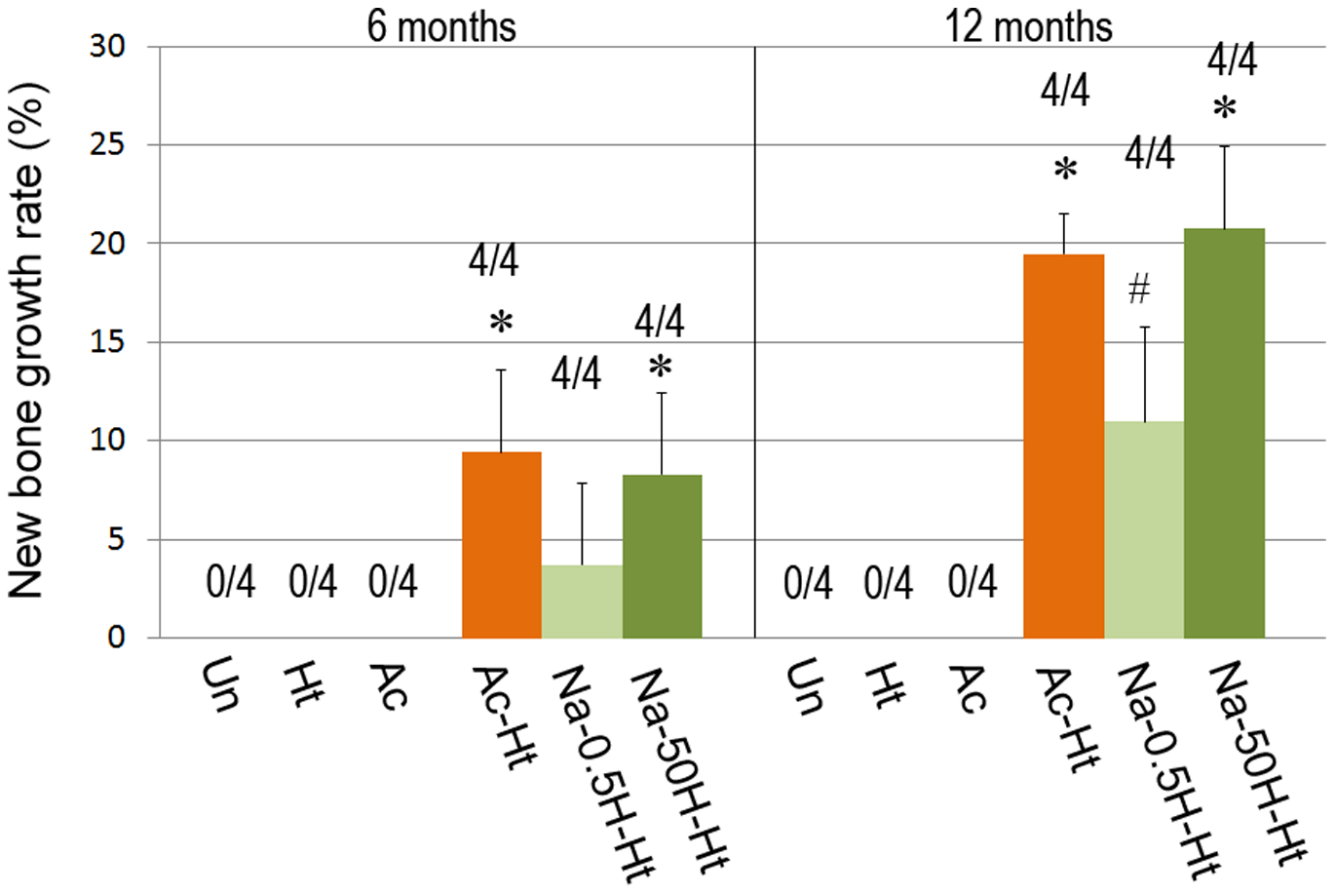
New bone growth rate on porous Ti metal specimens subjected to various treatments described in Table 1, evaluated 6 and 12 months after implantation. *: p<0.05 vs. Un, Ht, Ac and Na-0.5H-Ht. #: p<0.05 vs. Un, Ht and Ac. Un: Untreated, Ht: heat treated, Ac: Mixed acid treated, Ac-Ht: Mixed acid and heat treated, Na-0.5H-Ht: NaOH: NaOH, 0.5 mM HCl and heat treated, Na-50H-Ht: NaOH, 50 mM HCl and heat treated.

New bone growth rates of the porous Ti metal specimens are shown in Fig. 8. Bone formation was observed on three out of the six specimen types as shown in Fig. 8.

The porous Ti metal specimens treated by mixed acid and heat treatments (Table 1, Ac-Ht), or NaOH and concentrated acid treatment (Table 1, Na-50H-Ht) induced bone formation most actively. Samples that were NaOH, dilute acid and heat treated (Table 1, Na-0.5H-Ht) also induced bone formation, however the degree of bone formation was lower than that seen for the specimens treated by concentrated HCl.

A qualitative description of the ectopic bone formation or osteoinduction is included in Table 1.

## Discussion

All the Ti metals examined in the present study have the same porous structure with a porosity of 59% with interconnected 70–200 µm-pores (Fig. 3 (a)).

However, the surface texture and crystalline phases on the pore walls of the sample varied depending on the treatment used (Fig. 2 and 4).

The porous Ti metal specimens subjected to acid and heat treatments or specimens that were subjected to NaOH pretreatment followed by acid and heat treatments exhibited osteoinduction.

These osteoinductive specimens featured different surface textures and crystalline phases. Acid and heat treated specimens (Table 1, Ac-Ht) showed micrometer-scale roughness and rutile surfaces on the pore wall, while NaOH pretreated specimens (Table 1, Na-0.5H-Ht and Na-50H-Ht) featured nanometer-scale roughness with both anatase as well as the rutile on their pore walls (Fig. 2 and 4). Our results indicate that osteoinduction on porous Ti metal is not affected by a specific surface texture or specific crystalline phases present on the pore walls.

Among the three types of osteoinductive specimens, the contact angles of the NaOH pretreated specimens (Na-0.5H-Ht and Na-50H-Ht) were 11.2 (±1.8) and 27.6 (±3.8), respectively, while that of the Ac-Ht specimen was 58.8 (±2.3). The amount of bone formation was greatest in the Ac-Ht specimen, although its surface wettability was similar to the non-osteoinductive specimens (Un, Ht and Ac) (Table 1). There was therefore no clear correlation between surface wettability and the amount of osteoinduction.

The osteoinductive specimens described above all had positive zeta potentials and also showed high apatite-forming abilities in SBF, whereas all other non-osteoinductive porous Ti metals examined in the present study had almost zero zeta potential and no apatite-forming abilities in SBF, as shown in Table 1. This indicates that positive zeta potential and high apatite-forming ability in the porous Ti metals are determining factors for osteoinduction. For calcium phosphate based osteoinductive materials it has been speculated that ectopic bone formation was induced by an apatite layer formed on their surfaces in the living body [21]–[24]. Osteoinduction of our porous Ti metal specimens is also assumed to occur through the formation of an apatite layer in vivo. The high apatite-forming ability of our osteoinductive specimens in SBF indicates that an apatite layer could be formed on these porous Ti metal specimens in vivo.

The high apatite-forming abilities in SBF of our osteoinductive specimens might be attributed to their high positive zeta potentials. It has been shown that positively charged Ti oxide on the metal surface are important to the mechanism of apatite formation in SBF, as these preferentially adsorb negatively charged phosphate ions, and then positively charged calcium ions to form apatite on the metal surface, as already discussed in more detail elsewhere [10]. The effectiveness of the osteoinduction in the treated specimens correlated strongly with high positive zeta potential, as shown in Table 1. The treatments which induced high surface charge in the specimens, showed more active apatite formation and also induced ectopic bone formation more actively when implanted in dog muscles.

Zeta potential can also affect cell attachment and proliferation of osteoblasts. There are a number of papers reporting enhanced cell attachment and proliferation of osteoblast-like cells [25] and increased protein adsorption, which is necessary for cell attachment [26] on the Ti metal surfaces that have a positive zeta potential. This previous research suggests the positive charge on the specimens used in the present study may also contribute to enhanced osteoblast attachment and proliferation, consequently promoting bone formation in the porous area.

The nanostructure on the surface of the specimens is considered to have some effect on cell-implant interaction and tissue formation [27], [28]. Some studies have reported that human bone marrow mesenchymal stem cells differentiated in vitro into osteogenic lineage without any osteogenic supplements, exploiting predetermined nanostructures and geometries [29]–[31]. In the present study, the independent contribution of nanostructure to osteoinduction cannot be fully evaluated owing to the lack of a specimen with nanometer scale roughness, with no surface charge or apatite forming ability in vitro. Whereas the Na-0.5H-Ht and Na-50H-Ht specimens have similar nanometer-scale surface structures, Na-50H-Ht features a higher zeta potential than Na-0.5H-Ht. The amount of apatite formation on the surface was also higher for Na-50H-Ht than Na-0.5H-Ht. Similarly, the amount of induced bone was significantly higher in Na-50H-Ht than in Na-0.5H-Ht at 6 and 12 months. Although the nanostructure may have some contribution to the osteoinduction observed in these experiments, the comparison of Na-0.5H-Ht and Na-50H-Ht implies that surface charge is a determining factor for osteoinduction.

Based on these results, the following hypothesis can be given regarding the mechanism underlying the osteoinduction observed in this study. Positively charged Ti oxide on the metal surface forms an apatite layer on the surface, by first adsorbing negatively charged phosphate ions, and then positively charged calcium ions, as previously confirmed in vitro [10]. The formed apatite layer could then act as a physicochemical trigger for stem cells to differentiate into the osteogenic lineage.

Our finding that bone formation on porous Ti metal specimens can be enhanced through consecutive NaOH, HCl and heat treatments (Table 1, Na-0.5H-Ht and Na-50H-Ht) compared with only mixed acid and heat treatments (Table 1, Ht and Ac) which may also be of interest for the optimization of such materials for future clinical use.

## Conclusions

Porous Ti metal specimens with 59% connective 70–200 µm-pores exhibited osteoinduction in dog muscle when subjected to mixed-acid and heat treatments. The treatments produced pore surfaces with micrometer-scale roughness and rutile crystal phase. The untreated specimens did not exhibit any osteoinduction. Those that were heat treated had smooth pore wall surfaces with a rutile phase, and those subjected to a mixed-acid treatment had micrometer-scale surface roughness and pore walls composed of titanium hydride also did not exhibit any osteoinduction. The specimens subjected to consecutive NaOH, HCl and heat treatments, showed osteoinduction when implanted into the dog muscle. The osteoinduction results did not show any dependence on the surface characteristics of the specimens and could not be attributed to a specific surface texture of a specific crystalline phase on the pore walls.

However, all the osteoinductive porous Ti metals showed high positive zeta potentials and high apatite-forming ability in SBF. These results strongly suggest that ectopic bone formation is related to high positive surface charge and occurred via apatite formation on the metal surfaces in vivo.
